# Bilateral Atypical Femoral Fracture in a Bisphosphonate-Naïve Patient with Prior Long-Term Denosumab Therapy: A Case Report of the Management Strategy and a Literature Review

**DOI:** 10.3390/jcm13102785

**Published:** 2024-05-09

**Authors:** Kyle Auger, Jason Lee, Ian S. Hong, Jaclyn M. Jankowski, Frank A. Liporace, Richard S. Yoon

**Affiliations:** 1 Division of Orthopaedic Trauma & Adult Reconstruction, Department of Orthopaedic Surgery, Cooperman Barnabas Medical Center—RWJBarnabas Health, Livingston, NJ 07039, USA; kyle.auger@rwjbh.org (K.A.); jason.lee3@rwjbh.org (J.L.); ianshong93@gmail.com (I.S.H.); jaclyn.jankowski@rwjbh.org (J.M.J.); frank.liporace@rwjbh.org (F.A.L.); 2 Division of Orthopaedic Trauma & Adult Reconstruction, Department of Orthopaedic Surgery, Jersey City Medical Center—RWJBarnabas Health, Jersey City, NJ 07302, USA

**Keywords:** denosumab, atypical femoral fracture, bisphosphonate, bone antiresorptive therapy, subtrochanteric fracture

## Abstract

The benefits of denosumab as an antiresorptive therapy and in reducing fragility fractures are well documented. However, its association with atypical femur fractures (AFFs), especially in the absence of prior bisphosphonate use, remains poorly understood and warrants further investigation. This case report presents a rare instance of bilateral AFFs in a 78-year-old bisphosphonate-naïve patient with a history of long-term denosumab therapy for previous metastatic breast cancer. Management involved intramedullary nail fixation after initial presentation with a unilateral AFF and a recommendation to cease denosumab therapy. However, the patient subsequently experienced a contralateral periprosthetic AFF below a total hip implant 5 months thereafter and was treated with open reduction internal fixation. This case report highlights the critical need for orthopedic surgeons to maintain a high level of suspicion and vigilance in screening for impending AFFs, especially in patients with a prolonged history of denosumab therapy without prior bisphosphonate use. Furthermore, the growing report of such cases emphasizes the urgent need for comprehensive research aimed at refining treatment protocols that balance the therapeutic benefits of denosumab and its associated risks of AFFs.

## 1. Introduction

For decades, bisphosphonate therapy has been shown to be effective in reducing the risk of spine and non-spine fragility fractures in the setting of osteoporosis and metastatic bone disease [1,2]. While antiresorptive therapy has shown promising results, long-term use of these drugs has been associated with a higher risk of atypical femoral fractures (AFFs) [2,3,4]. This risk may be higher in patients with metastatic bone disease than in patients with osteoporosis [5]. Denosumab, a fully human monoclonal antibody, is an alternative option that prevents the formation, function, and survival of osteoclasts by binding to the receptor activator of nuclear factor-KB ligand (RANKL) and inhibiting the interaction of RANKL with RANK (the receptor on osteoclasts) [6,7].

A review article comparing denosumab and bisphosphonates showed that denosumab may have a more potent and long-lasting effect than bisphosphonate therapy, especially in postmenopausal osteoporotic females [8]. Several case reports have discussed this complication in osteoporotic and cancer patients using denosumab; however, most, if not all, of these studies are highly confounded by the former use of bisphosphonate and glucocorticoids, which have an established relationship with AFFs [6,9,10,11,12,13,14,15,16,17]. There is further uncertainty regarding the relationship between denosumab use and AFFs as four of these cases occurred after receiving only one dose of denosumab [11,12,13,15].

Here, we discuss a case report of a 78-year-old patient with a history of metastatic breast cancer status post mastectomy and chemotherapy presenting with both a subtrochanteric fracture and an impending atypical femoral diaphyseal fracture in the setting of long-term denosumab use without a history of bisphosphonate usage. The aim of this study is to add to the existing body of knowledge regarding this rare condition as well as to review the current evidence and provide the latest recommendations for orthopedic treatment and management.

## 2. Case Description

**Figure 1:**
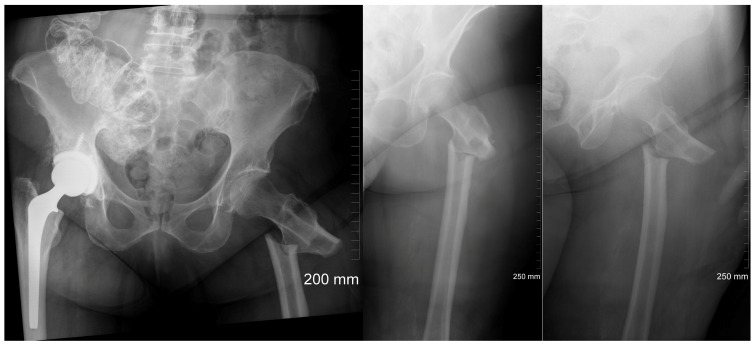
Radiographs demonstrating a transverse left subtrochanteric femur fracture with a medial spike in a 78-year-old female patient.

A 78-year-old female presented to our institution in May 2021 with a complaint of severe left hip pain after she heard a “crack” in her left hip while climbing stairs. The pain immediately was followed by inability to bear weight on the extremity, resulting in her resting on a step while waiting for assistance. She endorsed prodromal thigh pain to the left leg for 2 months prior to the incident as well as a past medical history of hypertension, hyperlipidemia, and breast cancer with bone metastases in 1994 and a post-mastectomy and post-chemotherapy-treatment status. Her surgical history included right mastectomy in 1994, a right total hip arthroplasty in 2014 following a pathologic hip fracture secondary to a fall, and a left total knee arthroplasty in 2018. Notably, at the time of presentation, the patient was on palbociclib, letrozole, and denosumab and had been taking denosumab since her hip fracture 7 years ago in 2014. However, she denied any previous bisphosphonate use. On examination, she was neurovascularly intact and had tenderness over the left groin and lateral thigh. X-ray and computerized tomography (CT) imaging were obtained demonstrating a transverse left subtrochanteric femur fracture with a medial spike (Figure 1). A traction pin was placed in the distal femur and 20 pounds of in-line skeletal traction was applied.

**Figure 2:**
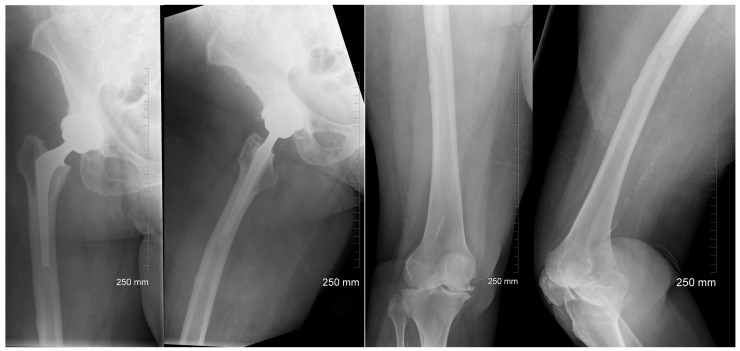
Radiographs of contralateral right femur demonstrating lateral cortical thickening distal to stem of femoral implant in 78-year-old female patient.

After the patient was stabilized and appropriate clearances were obtained, intramedullary nail fixation of the left femur was performed and histopathology showed no evidence of malignancy. She was made weight-bearing as tolerated and X-rays were obtained of the contralateral femur while in-house to evaluate for impending fracture. X-rays of the right femur demonstrated lateral beaking with cortical thickening distal to the femoral implant from her previous hip replacement surgery (Figure 2). Thus, magnetic resonance imaging (MRI) was additionally performed to exclude stress fractures, which was negative. Given the patient’s age, lack of clinical prodromal symptoms, and absence of a stress fracture on MRI, the decision was made to closely monitor the right femur with repeat radiographs during postoperative follow-up visits. However, she was made protective weight-bearing to the right lower extremity and discharged to a subacute acute rehabilitation facility with recommended cessation of her denosumab therapy.

She was subsequently seen in the office on postoperative week 2 to follow-up on her surgical care. Since being discharged from the hospital, she had been out of the rehabilitation center, walking with a rolling walker, and had advanced quickly with physical therapy. On physical examination, her sutures were intact, and her surgical incision was well healed with no signs of erythema or drainage. The traction pin site on the medial aspect of her distal femur showed some formation of granulation tissue around the area. She was non-tender over the left femur and exhibited good range of motion to the knee and ankle joint.

**Figure 3:**
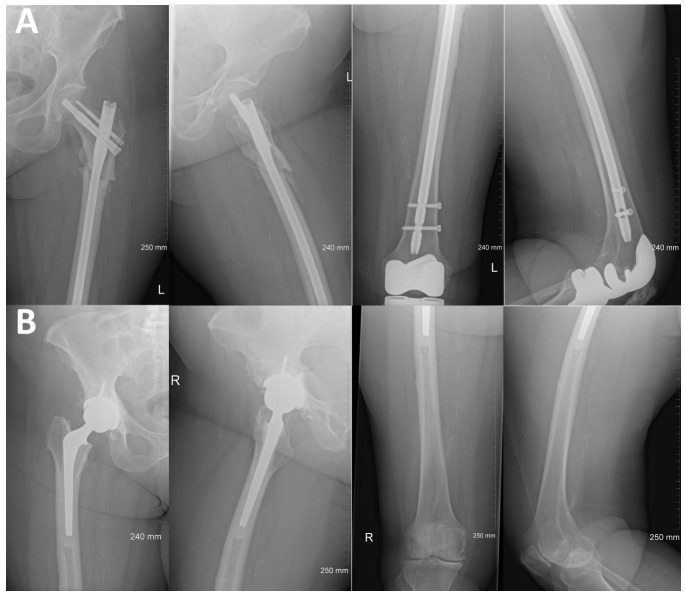
(**A**)—Three-month postoperative radiographs of left femur demonstrating delayed healing of subtrochanteric fracture with stable intramedullary nail fixation (**top**). (**B**)—Radiographs of the contralateral femur demonstrating lateral cortical beaking and thickening distal to the previous hip prosthesis (**bottom**).

The patient continued to do well until her 3-month postoperative follow up with no tenderness over the bilateral femurs and good range of motion to the knees. Radiographs of the left femur obtained at the time demonstrated delayed healing of the subtrochanteric femur fracture with stable orthopedic hardware and no signs of loosening (Figure 3A). Repeat contralateral femur radiographs continued to demonstrate lateral cortical beaking with an intact stable orthopedic implant and no evidence of fracture (Figure 3B). She had been maintaining protective weight-bearing precaution with a rolling walker to the right lower extremity as instructed, which remained asymptomatic. Due to her oncologist’s decision to continue denosumab therapy despite her impending fracture, discussion was had at this time for prophylactic fixation of her right femur with an intramedullary nail once she fully recovered from her left femur surgery.

**Figure 4:**
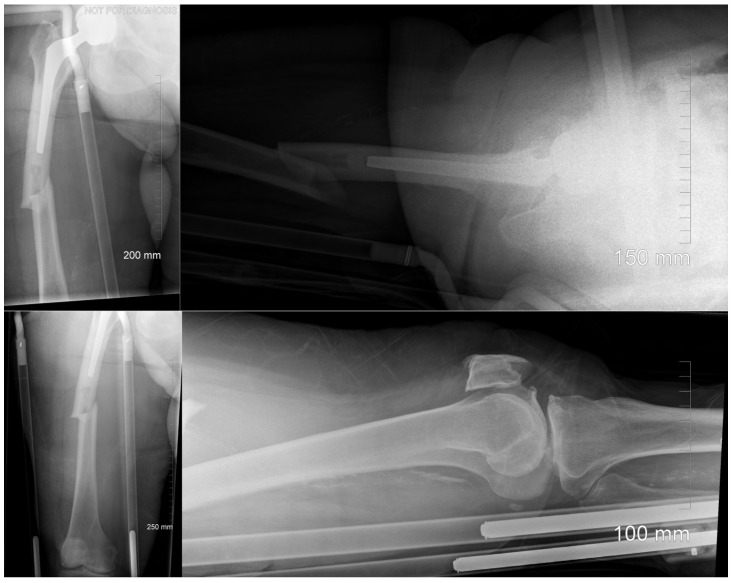
Radiographs demonstrating an acute atypical mid-shaft right periprosthetic femur fracture distal to the femoral stem (Vancouver Type C).

Unfortunately, 1 month after her most recent follow-up, in October 2021, the patient returned to the emergency department for right thigh pain while attempting to reach her sister at home who had fallen across the room. She stated she was ambulating with her rolling walker when she felt severe pain in her right femur during one of her strides. She denies falling but states she has been unable to bear weight with the affected leg following the incident. On examination, she was neurovascularly intact with tenderness over the mid-shaft femur region. X-rays were obtained demonstrating a well-fixated right hip replacement with an atypical mid-shaft femur fracture distal to the femoral stem (Vancouver Type C) (Figure 4). A traction pin was placed in the distal femur and 15 pounds of in-line skeletal traction was applied.

**Figure 5:**
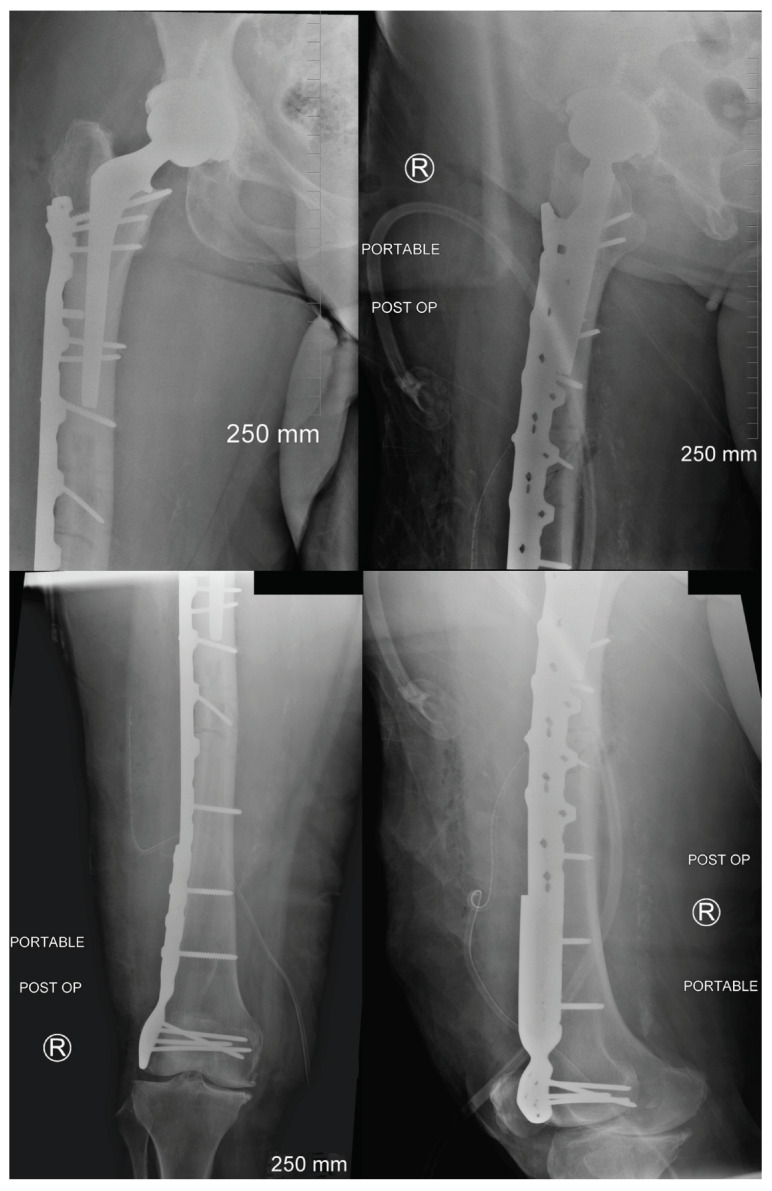
Immediate postoperative radiographs following open reduction and internal fixation with a lateral periprosthetic femoral plate.

After the patient was stabilized and appropriate clearances were obtained, the patient underwent open reduction internal fixation of the right femur with a lateral periprosthetic femoral plate (Figure 5). She tolerated the procedure well and was made flat-foot heel sliding weight-bearing on the right lower extremity and weight-bearing as tolerated on her left lower extremity and discharged to a subacute rehabilitation facility.

She remained in her rehabilitation facility with her right lower extremity flat-foot heel sliding weight-bearing precaution until her 6-week follow-up. She had been ambulating well with her restriction and was advanced to weight-bearing as tolerated to bilateral lower extremities at that time. Her surgical incisions were well healed at this point with good painless active and passive range of motion to the hips and knees. Radiographs obtained at this time demonstrated healing of the right femoral shaft fracture with intact orthopedic hardware.

During her 6-month follow-up for her right periprosthetic femoral shaft fracture, the patient reported mild right thigh pain only following rigorous physical therapy sessions but endorsed no left hip or thigh pain. At this point, she was utilizing a cane for long distance ambulation due to safety concerns and was very happy with her outcomes. Interval radiographs obtained demonstrate significant callus formation on the right femoral shaft fracture; however, there was a persistent fracture line noted within the left subtrochanteric femur that was unchanged from prior films. Now 11 months post left femur intramedullary nail, a CT scan was obtained for further evaluation which demonstrated a lack of callus formation at the fracture site and confirmed a diagnosis of nonunion. Discussion was had regarding exchange nailing with prophylactic open reduction internal fixation, for which the patient was ultimately scheduled 1 month later in May 2022.

**Figure 6:**
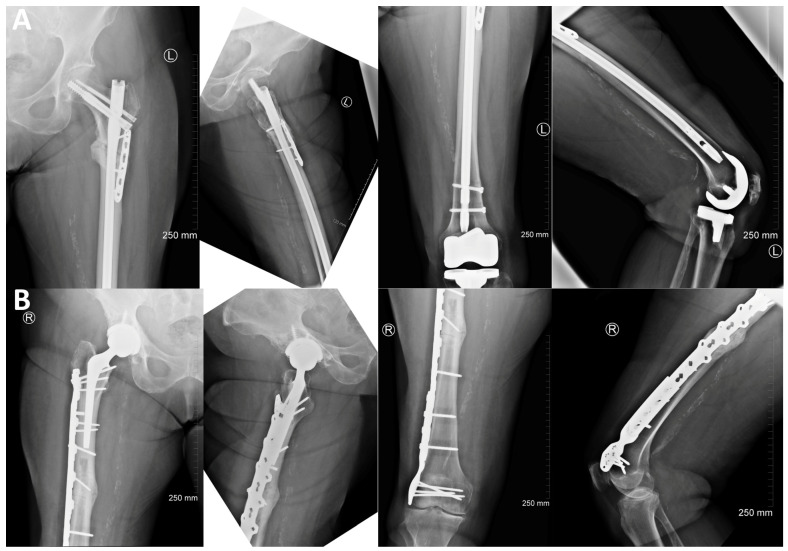
Most recent follow-up radiographs demonstrating healing of both femur fractures with no evidence of hardware failure: (**A**)—approximately 10 months following left femur nonunion surgery (**top**) and (**B**)—2 years after right atypical periprosthetic femoral shaft fixation (**bottom**).

She underwent the indicated procedure without complications, was made weight-bearing as tolerated, and was once again discharged to a subacute rehabilitation facility, where she was discharged home once independent. Throughout her follow-up appointments, she continued to progress with physical therapy without complaints of pain to the bilateral lower extremity fracture sites with evidence of bony healing during interval radiographs. On her last visit approximately 10 months following her left femur nonunion surgery and over 2 years after her right atypical periprosthetic femoral shaft repair, she was ambulating without assistive devices and had good painless passive and active range of motion to bilateral hips and knees. Radiographs demonstrated complete healing of both femur fractures with no evidence of hardware failure (Figure 6).

## 3. Discussion

Bisphosphonates and denosumab have been shown in randomized controlled trials to increase bone mineral density and decrease the incidence of fragility fractures within the geriatric and postmenopausal population [18,19,20,21,22]. Bisphosphonates function by inhibiting bone resorption by attaching to hydroxyapatite binding sites on bone. The embedded bisphosphonate is released as osteoclasts resorb bone, impairing their ability to further continue bone resorption [23]. Nitrogen-containing bisphosphonates achieve this by inhibiting farnesyl pyrophosphate synthase, a key regulatory enzyme in promoting osteoclast attachment to bone. Non-nitrogen-containing bisphosphonates on the other hand, replace the terminal pyrophosphate moiety of adenosine triphosphate to form a nonfunctional molecule that ultimately leads to osteoclast apoptosis [24]. This makes non-nitrogen-containing bisphosphonates (e.g., etidronate, tiludronate) much more potent antiresorptive agents than their nitrogen-containing counterparts (e.g., alendronate, neridronate, ibandronate, pamidronate, risedronate, and zoledronic acid)

Denosumab, a human monoclonal antibody, functions as an inhibitor of RANKL, which is essential for the regulation of bone metabolism. Physiologically, RANKL binds with RANK, a receptor found on osteoclasts, promoting osteoclast formation and subsequent bone resorption [25]. However, in perimenopausal females, there is evidence to suggest that RANKL levels are increased [25]. Denosumab, classified as an IgG2 immunoglobulin, binds with RANKL, thereby preventing its interaction with RANK and reducing osteoclast activity, and produces its effects in modulating bone remodeling processes.

In the FREEDOM trial, 7,868 postmenopausal women aged 60–90 years with osteoporosis were enrolled and randomized to receive either 60 mg injections of denosumab or placebo every 6 months for a duration of 36 months. The denosumab group showed a significant reduction in the risk of hip fractures, with a cumulative incidence of 0.7% in the denosumab group, versus 1.2% in the placebo group (hazard ratio, 0.60; 95% CI, 0.37 to 0.97; *p* = 0.04)—a relative decrease of 40% [22]. There were no fractures of the femoral shaft in the denosumab group and there were three such fractures in the placebo group (0.1%).

The efficacy of these drugs to prevent fragility fractures is without question, but their prolonged use has raised concerns for increased risk of atypical femur fractures (AFFs) within the community. The incidence of AFFs associated with bisphosphonate, as defined for treatments beyond 3 years, accounts for approximately 1.1% of all femoral fractures [26]. While bisphosphonates have long been associated with AFFs, there is emerging evidence suggesting that denosumab, despite its different mechanism of action, is not exempt from similar concerns of bisphosphonate use [27].

The incidence of AFFs associated with bisphosphonate use may vary but the number of published studies describing their association has led experts to conclude that long-term bisphosphonate use unequivocally contributes to the risk of suffering an AFF. The current literature on the underlying mechanism of AFFs offers several hypotheses, yet lacks a universally accepted pathogenesis for these fractures. Among the described mechanisms, Ettinger et al. proposed that long-term suppression of normal bone turnover by bisphosphonate reduces collagen plasticity and thus increases bone brittleness [28]. The result is the complete mineralization of cortical bone and a homogenous microstructure of bone. The combination of brittleness with a loss of heterogeneity allows for greater progression of microscopic cracks that can occur with usual physical activity. They suggest that the typical transverse fracture line is an indicator of the slow progression of a single microcrack progressing unimpeded due to failure of the usual mechanisms to bridge or deflect the crack.

Although the current literature on denosumab-associated atypical femur fractures is an area that requires further investigation, recent publications have highlighted the need for clinicians to carefully evaluate the long-term use of these therapies. Takahashi et al.’s retrospective analysis demonstrated that AFFs can occur in patients with bone metastasis treated with denosumab [4]. This current case report and other cases, reported by Go et al. [29] on bilateral AFFs in a 62-year-old female and Kumar et al. [30] on an 85-year-old female who after 5 years on denosumab developed a unilateral AFF, corroborate the fact that AFFs, although rare, can occur in patients that are bisphosphonate-naïve. Thus, these insights necessitate a proactive approach in monitoring and managing patients on denosumab, emphasizing the urgent need for further high-quality research to elucidate individual risk factors and develop comprehensive treatment guidelines.

These atypical fractures have been characterized by the American Society for Bone and Mineral Research (ASBMR) and can be identified by major and minor criteria [2]. Within the major criteria, they describe a transverse or short oblique fracture located between the distal border of the lesser trochanter of the femur to the proximal edge of the supracondylar flare with lateral cortical involvement and minimal or no precipitating trauma. These transverse fractures with an associated medial spike have become pathognomonic of atypical bisphosphonate femur fractures. The minor criteria include lateral cortical beaking, diaphyseal cortical thickening, prodromal groin or thigh pain, bilateral fractures, and delayed healing. The diagnosis of complete fracture is difficult to miss but it is essential for providers to remain vigilant and to maintain a high index of suspicion for incomplete or impending fractures within this population. Prodromal pain may be the only clinical sign of these impending fractures and can often go unrecognized or misdiagnosed, especially in weight-bearing patients.

In this case, despite the absence of prodromal symptoms and the recommendation to discontinue denosumab following the initial AFF, the denosumab therapy was continued by the oncology team given the patient’s previous history of metastatic breast cancer [31,32,33]. This highlights the necessity of an integrated interdisciplinary approach between the physician providing denosumab therapy and the treating orthopedic surgeon to balance the benefits of continued antiresorptive therapy against the risk of AFFs. While it cannot be definitively concluded that discontinuing denosumab would have prevented the subsequent fracture of the right femur, the possibility remains a significant concern. Additionally, following the initial fracture fixation of the left femur, the patient was allowed to be weight-bearing as tolerated on the operated limb and protected weight-bearing on the contralateral limb due to concerns of radiographic changes. However, there is also the possibility that the patient may have been inadvertently protecting her operated left femur, leading to increased stress on the right lower limb, which was under protected weight-bearing instructions. Although case reports such as this do not amount to causation, they highlight significant considerations. In patients that have been on a regimen of long-term denosumab therapy and demonstrate early radiographic changes preceding clinical prodromal symptoms, as observed in this case, the decision to implement prophylactic nailing should be considered carefully.

## 4. Conclusions

This case report highlights the critical need for orthopedic surgeons to maintain a high level of suspicion and vigilance in screening for impending AFFs, especially in patients with a prolonged history of denosumab therapy without prior bisphosphonate use. Furthermore, the growing report of such cases emphasizes the urgent need for comprehensive research aimed at refining treatment protocols that balance the therapeutic benefits of denosumab and its associated risks of AFFs.

## Data Availability

Data supporting the findings in the current study are reported in this article. The raw collected and reported data are available from the corresponding author on reasonable request.
